# The contribution of body perception to self-identity: an event-related potential study

**DOI:** 10.1093/scan/nsaf020

**Published:** 2025-02-27

**Authors:** Juanzhi Lu, Lars Riecke, Brenda E Ryan, Beatrice de Gelder

**Affiliations:** Department of Cognitive Neuroscience, Faculty of Psychology and Neuroscience, Maastricht University, Maastricht, Limburg 6200 MD, The Netherlands; Department of Cognitive Neuroscience, Faculty of Psychology and Neuroscience, Maastricht University, Maastricht, Limburg 6200 MD, The Netherlands; Institut d’Investigacions Biomèdiques August Pi i Sunyer (IDIBAPS), Carrer de Rosello 149, Barcelona 08036, Spain; Department of Cognitive Neuroscience, Faculty of Psychology and Neuroscience, Maastricht University, Maastricht, Limburg 6200 MD, The Netherlands

**Keywords:** body, face, self-identity, event-related potential, N2

## Abstract

This study used electroencephalography (EEG) and personalized avatars to investigate the neural mechanisms underlying personal identity perception. Compound avatar images combining participants’ own faces and bodies, as well as those of others, were generated from photographs. Participants underwent an embodiment training for each avatar type in a virtual reality environment, where they controlled the avatar’s actions during physical exercise tasks. Subjective assessments by participants confirmed a stronger identification with avatars representing their own identity compared to those representing others. Analysis of event-related potentials (ERPs) evoked by viewing the avatar revealed that avatars representing the participants’ self-identity elicited weaker N2 and P1 responses compared to avatars representing other identities. No significant effects on N170 responses were observed. Control conditions utilizing avatars with modified body characteristics confirmed that the reduction in N2 amplitude was specifically related to identity perception rather than variations in visual body size. These findings suggest that the perception of self-identity occurs rapidly, within ∼200 ms, indicating the integration of visual face and body information into identity representation at an early stage.

## Introduction

Understanding and processing someone’s identity is fundamental to our social interactions. We effortlessly recognize familiar individuals and distinguish between acquaintances and strangers. But we care just as much about our own identity. We have a unique ability to recognize our own physical appearance, often scrutinizing our own facial and bodily features when faced with a mirror. Previous research on self-perception has primarily focused on facial stimuli to investigate the behavioral and neural processes involved in identity perception and recognition (Sui et al. 2009, Maister et al. 2013, Gonzalez-Franco et al. 2016). The significance of self-face recognition has long been debated and recent meta-analyses confirm that individuals identify their own face more quickly than others’ faces (Bortolon and Raffard 2018).

EEG studies exploring the neural basis of self-facial identity perception have identified that the N2 component, an event-related negativity occurring at ∼200 ms, is associated with self-identity and self-relevance (Tanaka et al. 2006, Pierce et al. 2011, Woźniak et al. 2018, Muñoz et al. 2020, Bola et al. 2021). Woźniak et al. (2018) found that self-associated faces elicit smaller N2 amplitudes than faces associated with others, such as friends or strangers, indicating an early neural process for self-face perception. Consistent with this, Bola et al. (2021) demonstrated that self-face stimuli, but not other-face stimuli, elicit the N2pc component within 200 ms. Muñoz et al. (2020) found that personal objects elicited lower N2 amplitudes compared to nonpersonal objects, further supporting the role of the N2 in self-relevance processing. These findings collectively suggest that the attenuation of N2 associated with self-identity from self-relevant stimuli, such as one’s own face, occurs rapidly within ∼200 ms.

Much less is known about self-perception based on the body and the combined perception of the self-face and body. The importance of body perception for self-perception is illustrated by clinical studies showing that body perception-based self-awareness can sometimes lead to dissatisfaction with our physical appearance, contributing to conditions like eating disorders and body dissatisfaction, as evidenced by clinical studies (Karazsia et al. 2017). But despite its importance, the neural mechanisms underlying self-body perception remain poorly understood. To date, only a few studies have delved into the perception of self-identity from bodies or used personalized body images. In a functional magnetic resonance imaging study, researchers utilized full-body photos of participants clad in dark bathing suits and found that photos conveying self-identity triggered responses in the extrastriate, parietal regions, and middle frontal gyrus in the right hemisphere (Hodzic et al. 2009). An event-related potential (ERP) study employed participant photos, presenting naturalistic, enlarged, and reduced body images to investigate the perception of self-body versus other-body under various attention tasks (Uusberg et al. 2018). Their findings indicate that the distinction between self-body and other-body influences the N170 component, renowned for its role in the initial stages of face or body perception (Stekelenburg and de Gelder 2004). However, our comprehension of the neural dynamics underlying the perception of self and other identities based on body images remains limited.

A related inquiry concerns the relative significance of the face and body. Based on the literature where studies on self-facial identity largely outnumber those on self-body identity, one might assume that face identity drives self-recognition, with the body playing only a minor role. If so, the presence of the personalized body would not make a difference to self-identity perception. However, studies on the perception of emotional expressions have shown that body expression can influence how facial emotion is processed already in the early processing stages as seen in the EEG on the P1 (Meeren et al. 2005). Thus, it is conceivable that face and body information are integrated in early neural processes to form a self-identity percept.

To test this hypothesis in the current study, we explored the neural basis of self-identity perception using avatars synthesized from personalized face and body images. To enhance the participants’ sense of body ownership of the avatar, we devised an embodiment task employing a virtual reality (VR) environment for physical exercise. Participants faced a virtual mirror displaying their avatar and engaged in physical activities (e.g. moving their arms to catch bubbles and reaching for crystals). This VR session aimed to bolster the participants’ identification with the avatar and their sense of body ownership. During the experiment proper, participants viewed various categories of avatars and performed an oddball task, while EEG was recorded. EEG provides exceptional temporal precision, offering millisecond-level resolution, which is ideal for investigating the temporal dynamics of self-identity processing. Building upon previous findings with personalized face images and bodies, we anticipated that the perception of self-identity based on avatars with their own face and their own body would modulate N2 and N170 responses compared to the perception of other-identity avatars. As previous studies have linked P1 to sensory input such as image size (Busch et al. 2004, Pfabigan et al. 2015), we also included avatars with altered body sizes and analyzed P1 responses to control for visual image size differences between self and other avatars.

## Materials and methods

### Participants

Twenty-nine healthy participants [aged 18–24 years, mean = 19.9; standard deviation (SD) = 1.7; one left-handed] were recruited from the female student population at Maastricht University. All participants had a normal body mass index (BMI = 18.5–24.9), normal or corrected-to-normal vision, and no history of brain injury, psychiatric disorders, or current use of psychotropic medication. Before the experiment, participants provided written consent. They received compensation of 7.5 Euros or one credit point per hour for their participation. The Ethics Committee of Maastricht University approved the study, and all procedures adhered to the principles outlined in the Declaration of Helsinki.

### Experimental design and stimuli

Personalized avatars for each participant were created from frontal photographs of the participant showing a full frontal view of body and face with a neutral posture and expression. These images served as the basis for generating individualized avatar body trunks, closely matching the participant’s body shape, and avatar faces that accurately replicated the participant’s facial features. We refer to these components as “self” or “own.” Additionally, we generated avatar body trunks and avatar faces from images of averaged Dutch body and face profiles. We refer to these components as “other.” We digitally manipulated all body images to generate additional, heavier-weighted versions of avatars with big body trunks.

**Figure 1.**
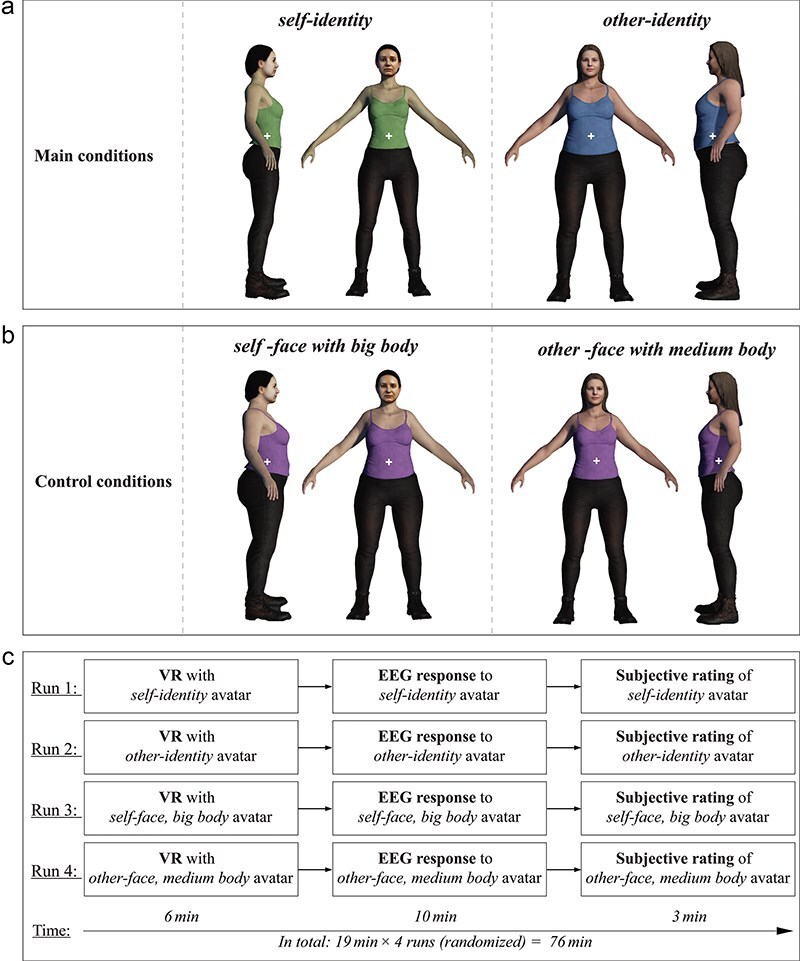
Experimental design. Four example avatars are shown (one per condition). (a) It depicts the main conditions, featuring the participant’s self-identity avatar (composed of the participant’s own face and own body) on the left, and the other-identity avatar (composed of others’ face and body) on the right. These avatars were expected to elicit strong self-identity percepts and a clear other-identity percept, respectively. (b) It depicts the control conditions, featuring an avatar composed of the participant’s own face with a big body and another avatar composed of other-face with a medium body. The latter avatars were expected to elicit body-size effects in the absence of strong identity differences. Avatar orientation and shirt color were pseudorandomly varied within each condition. This figure is used with permission of the participant resembled in the self-identity condition images. (c) It depicts the experimental protocol. The order of runs was randomized across participants.

To elicit a full self-identity percept, we combined the participants’ own avatar face with the participants’ own avatar body. Similarly, to elicit an other-identity percept, we combined the other avatar face with the other, big body trunk avatar. Examples of these two main avatars are illustrated in Fig. 1a. As these avatars differed in both (putative) identity percept and body-trunk size, we included two altered-body conditions that served to assess potentially confounding body-size effects in the absence of strong identity differences. These control avatars combined the participants’ own avatar face with the participants’ big body trunk avatar (Fig. 1b, left) and the other avatar face with the other, medium body trunk avatar (Fig. 1b, right). All avatars were generated using the Character Creator 3 software.

In order to increase the naturalness and variability of the avatar images during the EEG recordings, we pseudorandomly varied the color of the avatar’s T-shirt (blue, purple, or green) and the avatar’s orientation (frontal, 90° left, or 90° right), resulting in nine unique avatar images for each condition. The avatar was presented on a transparent background (size: 700 × 1000 pixels) covering ∼3.2° × 4.5° of participants’ visual angle in the experiment. In order to make participants focus on the whole body, we centered a white fixation cross onto the avatar’s body near the waist (Fig. 1).

### Experimental procedure

Before admitting participants to the EEG study, their ability to recognize and distinguish their own avatar image from other avatar images was assessed with an online pre-questionnaire. When participants passed this initial screening, they were invited to the lab, where the experimenters introduced the experiment, prepared the EEG (see the “EEG acquisition” section), and seated them in a comfortable chair. Participants underwent four runs of the experiment lasting in total ∼1 h. Each run consisted of three parts that directly followed each other: VR task, EEG task, and post-questionnaire. In the VR task, participants performed a behavioral task in a VR environment while being embodied in an avatar corresponding to one of the four conditions. The purpose of this task was to create embodiment in the current avatar condition. In the subsequent EEG task, participants performed a behavioral task on 2D images of the same avatar as in the VR task; the images were shown on a computer screen, while EEG was recorded. In the subsequent post-questionnaire, participants answered three questions related to the VR and avatar. The order of runs (i.e. conditions) was randomized across participants. Details about pre-questionnaire, VR task, EEG task, and post-questionnaire are described in the next subsections.

#### Pre-questionnaire

To assess whether participants could recognize their personalized self-identity avatar and distinguish it from other avatars, we presented them with images of the self-identity avatar and images of the control avatar composed of other-face with medium body. We asked participants to rate whether the avatar looked similar to themselves (“similarity”) and whether they could recognize the avatar (“recognizability”) on a seven-point Likert scale (1: not agree at all, 7: strongly agree) before the experiment (Table 1, light shading).

**Table 1.** Pre-questionnaire and post-questionnaire.

Questionnaire	Aim	Question	Rating
Pre-questionnaire	Similarity	The avatar looks like me.	1 (do not agree at all) to 7 (strongly agree)
	Recognizability	I can recognize myself in this avatar.	1 (do not agree at all) to 7 (strongly agree)
Post-questionnaire	Embodiment	I felt that the virtual body I saw when I was in VR was my own body.	1 (do not agree at all) to 7 (strongly agree)
	Identification	The body I saw physically looked like me.	1 (do not agree at all) to 7 (strongly agree)
	Liking	How did you feel about the avatar?	1 (do not like it at all) to 7 (I like it very much)

#### VR embodiment task

The VR scenario was programmed in Unity Software in collaboration with Virtual Bodyworks Company (Barcelona, Spain). Participants stood upright wearing a VR headset (Oculus Quest 2). In the VR environment, they viewed a given avatar (depending on the experimental condition) in a mirror from a first-person perspective and performed a target catching task. This task required them to manually catch bubbles and reach crystals through different circles in the VR by executing corresponding arm movements in the physical world.

#### EEG task

In the EEG experiment, each trial consisted of the presentation of an avatar image for 1000 ms, preceded by a fixation cross for 500 ± 100 ms. To make participants focus on the avatar, an oddball task was used. This task required them to press a button when they detected a deviant avatar, which consisted of different physical features (hair style, skin color, and facial features). The deviant avatars were also presented in nine variations (3 shirt colors × 3 orientations) and they never occurred on two consecutive trials. We instructed participants to press the button when they saw the deviant avatar, and we emphasized response accuracy (we did not encourage response speed; therefore, we did not analyze reaction time below). In each run, each standard image was repeated 42 times and each deviant image was repeated 4 times, resulting in 378 standard trials and 36 deviant trials per run (i.e. condition). Each EEG run lasted ∼10 min.

#### Post-questionnaire

To assess the participants’ subjective feelings about their embodiment, avatar identification, and avatar liking (Marchiondo et al. 2015, Mello et al. 2022), we asked them three questions after each EEG run. These questions are described later (Table 1, dark shading).

### EEG acquisition

EEG data were recorded using an elastic cap with 64 electrodes placed according to the international 10-20 system and sampled at a rate of 1000 Hz (BrainVison Products, Munich, Germany). Electrode Cz was used as the reference during recording, and the forehead electrode (Fp1) was used as a ground electrode. Two electrodes were used to measure the vertical and horizontal electrooculogram. The remaining 58 electrodes included FPz, AFz, Fz, FCz, CPz, Pz, POz, Oz, AF7, AF8, AF3, AF4, F7, F8, F5, F6, F3, F4, F1, F2, FC5, FC6, FC3, FC4, FC1, FC2, T7, T8, C5, C6, C3, C4, C1, C2, TP9, TP10, TP7, TP8, CP5, CP6, CP3, CP4, CP1, CP2, P7, P8, P5, P6, P3, P4, P1, P2, PO7, PO8, PO3, PO4, O1, and O2. Impedances for reference and ground were maintained <5 kOhm and for all other electrodes <10 kOhm.

### EEG data preprocessing

EEG data were preprocessed and analyzed using FieldTrip version 20220104 (Oostenveld et al. 2011) in Matlab R2021b (MathWorks, USA). Recordings were first segmented into epochs from 500 ms pre-stimulus (the avatar image) to 1500 ms post-stimulus and then filtered with a 0.3–30-Hz band-pass filter. EEG data at each electrode were re-referenced to the average of all electrodes. Artifact rejection was done using independent component analysis (logistic infomax ICA algorithm, Bell and Sejnowski 1995); on average, 1.56 ± 0.70 (mean ± SD) components were visually identified as artifacts and removed per participant. Moreover, single epochs during which the EEG peak amplitude exceeded 3 SD above/below the mean amplitude were rejected. On average, 270.03 ± 39.02 trials were preserved and statistically analyzed per participant.

### ERP analyses

Epochs from 200 ms before until 1000 ms after stimulus onset were extracted from the preprocessed data. Baseline correction was applied and involved subtracting the average amplitude in the baseline interval (−200 to 0 ms) from the overall epoch. Trials were averaged for each experimental condition, resulting in ERPs used for further statistical analyses, which were performed using IBM SPSS Statistics 27 (IBM Corp., Armonk, NY, USA). Only trials comprising standard stimuli were analyzed. We spatially separated the EEG electrodes into an occipital cluster (POz, Oz, PO3, PO4, O1, and O2), temporal cluster (P7, P8, TP7, TP8, CP5, CP6, P5, and P6), and frontal cluster (F1, F2, Fz, FC1, FC2, and FCz) and averaged the channels within each cluster. For each cluster, we pooled all conditions and visually identified a prominent ERP component based on visual inspection of the overall ERP waveform, topographical distribution of grand-averaged ERP, and previous studies (Xu et al. 2017, Muñoz et al. 2020, Lu et al. 2023). The identified ERP components and their associated time windows are as follows: P1 (80–130 ms) in the occipital cluster; N170 (140–190 ms) in the temporal cluster; and N2 (200–250 ms) in the frontal cluster. The mean amplitude was computed as the average of all electrodes within each cluster within the specific time window.

Outliers (data points further than 3 SD away from the mean) were rejected; this affected one participant’s dataset in the analyses of N170. All other datasets were normally distributed as assessed with the Shapiro–Wilk test. Paired *t*-tests were applied to compare the mean ERP amplitudes (or differences between these amplitudes) across conditions. Statistical results were considered as significant given a *P*-value of <.05.

## Results

### Pre-questionnaire results

Rating scores for similarity and recognizability were significantly larger for the self-identity avatar than the control avatar, which was composed of other-face with medium body (similarity: self-identity avatar: 5.45 ± 1.40, altered-body avatar: 2.72 ± 1.46, *t*(28) = −10.21, *P *< .001; recognizability: self-identity avatar: 5.86 ± 1.27, altered-body avatar: 2.59 ± 1.48, *t*(28) = −9.96, *P *< .001). These observations indicate that participants could successfully identify and discriminate their personalized avatar.

### Post-questionnaire results

**Figure 2.**
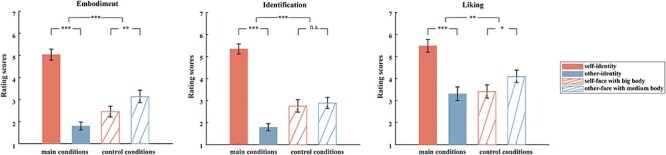
Post-questionnaire results. Means and standard error (SE) of rating scores for embodiment (left), identification (middle), and liking (right) per condition. ***P < .001, **P <0.01, *P < .05, n.s.: non-significant.

The rating scores of embodiment showed a significant difference between self-identity and other-identity conditions (*t*(28) = 13.15, *P* < .001). Specifically, the embodiment scores for self-identity (5.03 ± 1.37) were substantially higher than those for other-identity (1.79 ± 0.98). Additionally, a significant difference was observed between the control conditions (*t*(28) = 3.58, *P* = .001), with higher embodiment scores for other-faces with medium bodies (3.13 ± 1.55) than self-faces with big bodies (2.44 ± 1.32). Notably, the difference between main conditions was significantly larger than that between control conditions [*t*(28) = 7.23, *P* < .001, difference between main conditions (self-identity minus other-identity): 3.24 ± 1.33, difference between control conditions (other-faces with medium bodies minus self-faces with big bodies): 0.69 ± 1.04]. Applying the same analyses to identification scores and liking scores yielded results qualitatively identical to those mentioned earlier, except that the identity had no significant effect on identification scores in the control conditions. Overall, the subjective rating data indicate that participants identified more strongly with the self-identity avatar than the other-identity avatar (Fig. 2).

### Event-related potentials

#### Effect of self- versus other-identity perception

**Figure 3.**
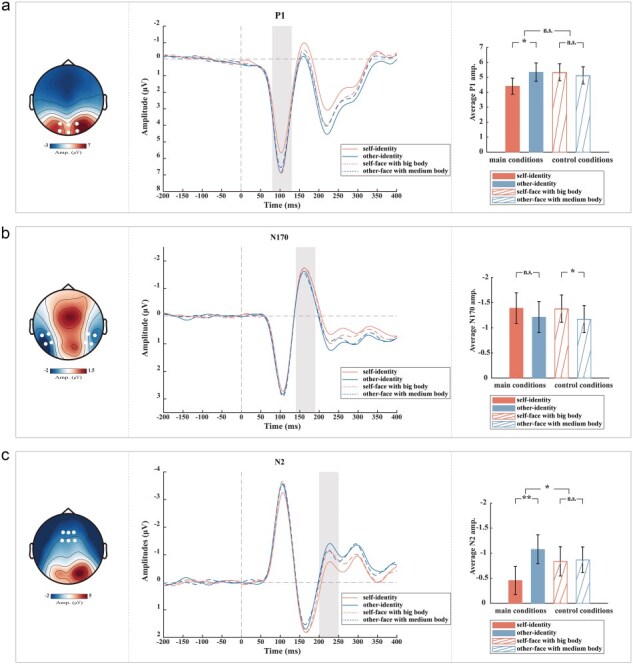
(a–c) EEG results. Grand averaged ERPs are depicted per condition for P1, N170, and N2 components separately (middle). The shaded rectangle visualizes the time window from which the average ERP amplitude was extracted. The highlighted white dots on the topographic map (left) represent the electrodes from which the grand-averaged ERP for each component was extracted. Bar plots (right) illustrate the mean and standard error (SE) across participants of each component’s amplitude per condition. **P < .01, *P < .05, n.s.: non-significant.

To investigate the temporal dynamics of brain activity during the perception of self-identity versus other-identity, we compared ERPs elicited by viewing participants’ own avatar (self-identity condition) versus another avatar (other-identity condition). Figure 3a shows that the self-identity condition (4.40 ± 2.97 µV) elicited smaller P1 amplitudes than the other-identity condition (5.34 ± 3.36 µV). This observation was confirmed by a significant effect of identity on P1 amplitude (*t*(28) = −2.09, *P* = .046). In line with our hypothesis, we found a similar effect of identity on N2 (*t*(28) = 2.82, *P* = .009; self-identity: −0.46 ± 1.54 µV, other-identity: −1.08 ± 1.59 µV). We found no effect on N170 (*t*(27) =  −1.20, *P* = .240).

#### Effect of identity perception versus effect of body size

To exclude that the observed effect on P1 and N2 reflected merely visual body differences (rather than identity, i.e. the integration of the own face with the own body), we compared the other-face, medium body avatar versus the self-face, big body avatar. Compared to the main conditions, these control conditions significantly reduced overall identity differences (see the “Post-questionnaire results” section) while preserving differences in physical body size. Unlike the main results above, the comparison of these control conditions yielded no effect on P1 or N2 (P1: *t*(28) = −1.45, *P* = .158; N2: *t*(28) = −0.24, *P* = .815), but on N170 (*t*(27) = 2.67, *P* = .013; other-face with medium body: −1.17 ± 1.46 µ, self-face with big body: −1.38 ± 1.46 µV), suggesting that the main results above do not reflect merely visual body differences.

To confirm this notion, we statistically compared the observed effect of identity perception versus the observed (null) effect of body size. We found a significant difference only for the N2 component, such that the difference between the main conditions was larger than the difference between the control conditions (*t*(28) = 2.55, *P* = .017; self-identity minus other-identity: 0.62 ± 1.19 µV; other-face with medium body minus self-face with big body: −0.03 ± 0.72 µV). This result suggested that the effect on N2 can be attributed to identity perception, rather than body size. We found no significant difference for P1 (*t*(28) = −1.64, *P* = .113), suggesting that the effect on P1 cannot be attributed to identity perception alone, but also to body size.

#### Comparison of self-identity condition versus control conditions

We further explored differences between the self-identity condition and the control conditions, for which participants gave reduced identification ratings as stated earlier (see the “Post-questionnaire results” section). Consistent with the identification-rating results, we found that the self-identity condition elicited smaller N2 amplitudes than the self-face with big body (*t*(28) = 2.13, *P* = .042) and the other-face with medium body (*t*(28) = 2.04, *P* = .051). Applying the same analyses to P1 yielded a significant difference to the self-face with big body (*t*(28) = −2.13, *P* = .042), but not to the other-face with medium body (*t*(28) = −1.73, *P* = .095), further supporting the above notion that P1 was modulated by body rather than identity. Overall, the N2 responses and identification ratings showed a strikingly similar pattern across conditions (Fig. 3c versus Fig. 2, middle). These observations further support the notion that N2 was modulated by identity perception, whereas P1 was affected more by visual body size.

## Discussion

We investigated neural responses to self-identity, other-identity, and altered-body avatar images. In line with our hypothesis on the importance of body identity, we found a clear effect of self-identity based on the face and the body on the N2 component: avatars with whom participants could identify more strongly elicited significantly smaller N2 amplitudes than avatars with whom participants identified less. Importantly, this suppressive effect on N2 was caused by self-identity as defined “jointly by the face and the body”: self-face avatars elicited smaller N2 amplitudes only when the avatar’s body also resembled the participant. In contrast, the earlier P1 component showed an effect of visual body size in the main condition (and a corresponding trend in the control condition): avatars with bigger bodies elicited larger P1 amplitudes than avatars with smaller bodies. We further observed that self-face avatars elicited stronger N170 amplitudes than other-face avatars; however, like the P1 results, this observation showed no systematic link to self-identity.

### Effect of face and body on subjective ratings of identity, embodiment, and liking

Behavioral results showed that participants identified more strongly with avatars that contained both their own face and body compared to avatars whose faces and bodies were not generated from themselves. Avatars with a face and/or a body that did not resemble the participant received significantly lower identification ratings. Surprisingly, the self-face avatar with the big body was rated similarly as the other-face avatar with the medium body. This suggests that the presence of facial identity alone is insufficient for triggering self-perception, regardless of body self-identity. Instead, self-identity seems to be perceived based on a combination of the body and the face. In line with this, it has been shown that a task-irrelevant body expression can influence face identity (Stock and Gelder 2014). Our finding contrasts with the traditional emphasis on the role of the face for identity perception. A review of previous studies about combined face–body perception for person identity found that observers tend to attend selectively to the face and ignore the body, unless the facial information is insufficient to provide identity information (Hu et al. 2020). But previous studies did not use personalized avatar images that the participants had become acquainted with through VR embodiment. Still, we cannot exclude that the instructions to fixate on the center of the image (which was in facet on the body) may have contributed to a reduction in the impact of the face component.

Unlike the identification ratings, participants’ ratings of embodiment and liking showed a significant difference between the control conditions. Participants embodied and liked more the other-face avatar with the medium body than the self-face avatar with the big body. The main conditions showed a similar effect, with higher ratings for the self-face, self-body avatar than the other-face avatar with the big body. Together, this shows that participants embodied and liked more the medium (more self-like) body than the bigger body, regardless of face identity. Face identity alone may not be not as important for embodiment and liking as one may have expected.

Overall, these subjective rating data indicate that embodiment and liking seem to be driven primarily by the perception of the body, whereas identification may be driven more by a combination of face and body perception. However, the three measures turned out to yield overall very similar results, suggesting that they measure partially the same construct.

### Effect of self-identity on N2

Our main finding is that the perception of self-identity based on the face and the body reduces N2 responses. In the main conditions, we observed that self-face, self-body avatars elicited smaller N2 amplitudes than other-face, big-body avatars. In contrast, in the control conditions, we found no significant N2 difference between self-face, big-body avatars and other-face, medium-body avatars. Moreover, N2 responses showed a significantly larger difference between the two main conditions than between the two control conditions. These observations rule out that the effect observed in the main conditions reflects body-size differences and instead support a genuine effect of self-identity perception. The latter is further supported by our observation that the N2 responses followed a pattern across conditions that closely resembled participants’ identification ratings. This strongly suggests that both measures reflect the same phenomenon.

Our finding that self-identity perception reduces the N2 amplitude is consistent with results from previous studies that showed N2 reduction related to self-relatedness, familiarity, and ownership (Carver et al. 2006, Woźniak et al. 2018, Muñoz et al. 2020, Kotlewska et al. 2023). Noteworthy, the reduction of N2 has been attributed to a rather automatic process (Todd et al. 2008), suggesting that self-identity perception as reflected by N2 suppression might emerge with relatively little cognitive effort.

We further observed that self-identity avatars elicited smaller N2 amplitudes than avatars combining the self-face with a big body or the other-face with a medium body. This indicates that the N2 reduction is sustained by the identity of not only the face but also the body. Previous studies reported that self-faces elicit smaller N2 amplitudes than other-faces including famous faces (Kotlewska et al. 2023), indicating that N2 is sensitive to self-identity. While consistent with this interpretation, our results further reveal that when a body is present, its identity “needs to fit that of the face.” Thus, our findings extend the previous N2 findings by showing that the previously observed suppressive, self-face related effect on N2 is largely attenuated when the self-face is combined with a big body. This further suggests that the N2 suppression does not reflect the self-face alone, but the self-identity as defined by the combination of face and body.

It should be noted that the observed N2 reduction might reflect a process that is more general than self-perception alone. Muñoz et al. (2020) found significantly smaller N2 response to personal objects than nonpersonal objects (Muñoz et al. 2020). Similarly, unfamiliar objects have been found to elicit more negative N2 responses than familiar objects (Carver et al. 2006). Therefore, the N2 reduction might reflect more general percepts of personal identity and/or familiarity.

### Effect of body size on P1

We found that the self-identity avatar elicited significantly smaller P1 amplitudes than the other-identity avatar with the big body. A similar trend was observed in the control conditions: other-face avatars with medium-sized body elicited smaller P1 amplitudes than self-face avatars with a big body. Therefore, unlike the N2 results, the effect on P1 reflected visual differences in body size, rather than self-identity. Several previous studies have shown that the P1 component is related to the visual stimulus size (Busch et al. 2004, Niu et al. 2008, Pfabigan et al. 2015, Schindler et al. 2019), with larger-sized stimuli eliciting larger P1 amplitudes. Our results confirm these previous findings and further reveal that the size sensitivity of P1 persists even when visual input elicits distinct identity percepts.

### Effect of face, but not self-identity, on N170

In contradiction with our hypothesis, we found no clear effect of self-identity (face and body) on the N170 component. We observed that self-face avatars with big bodies elicited significantly larger N170 amplitudes than other-face avatars with medium bodies. A similar trend was observed in the main conditions: self-face avatars with self-bodies elicited slightly larger N170 amplitudes than other-face avatars with big bodies. Therefore, unlike the N2 results, the effect on N170 reflected differences between self-face and other-face, with little relation to the body or overall identity. This interpretation is in line with previous findings showing larger N170 responses to self-faces versus faces of friends or strangers (Keyes et al. 2010).

In summary, our data highlight the critical role of body expression in perceiving self-identity. Interestingly, participants embodied and liked medium bodies (closer to their own body size) more than bigger bodies, regardless of the face-identity. Participants preferred the medium body over the bigger body even when the former had someone else’s face, suggesting that body size plays a significant role in identity perception. These findings have important social and clinical implications, particularly for understanding attitudes toward obesity. Moreover, VR technology could serve as a tool to help individuals with obesity engage in physical exercise or training by allowing them to embody avatars of varying body sizes in realistic, immersive environments.

Drawing these conclusions from our study requires some cautionary remarks. First, to eliminate potential confounds related to gender matching between participants and the female avatars, we only recruited female participants, implying that our findings may not generalize to males. Second, our sample consisted exclusively of young adults and Caucasian participants, and further research is needed to understand to which extent cultural differences (e.g. among Asian populations) might affect our results. Finally, the individualization of the stimuli came with the drawback that participants’ bodies could not be systematically matched to the control condition. Although participants’ bodies had normal BMI, some were bigger, and others were smaller than the Dutch medium one (which also had normal BMI). Consequently, size might have affected the individual results, but a systematic size effect at the group level is unlikely.

## Conclusion

The perception of self-identity (as defined jointly by the face and the body) leads to a reduced amplitude of the N2. This indicates that self-identity perception emerges rapidly in the brain within 200 ms, suggesting that at this stage visual face and body information have been integrated into person identity. Conversely, image size and face identity are processed in earlier stages as reflected by increases in P1 and N170 responses, respectively.

## Data Availability

The data underlying this article will be shared on reasonable request to the corresponding author.
